# Working Environment, Personal Protective Equipment, Personal Life Changes, and Well-Being Perceived in Spanish Nurses during COVID-19 Pandemic: A Cross-Sectional Study

**DOI:** 10.3390/ijerph19084856

**Published:** 2022-04-16

**Authors:** Segundo Jiménez-García, Alba de Juan Pérez, Rosa M. Pérez-Cañaveras, Flores Vizcaya-Moreno

**Affiliations:** 1 Infectious Diseases Unit, Elda University Hospital, 03600 Alicante, Spain; segundo.jimenez@ua.es; 2 Preventive Medicine and Public Health Service, San Juan University Hospital, 03500 Alicante, Spain; alba_djp@ua.es; 3 Clinical Nursing Research Group, Department of Nursing, Faculty of Health Sciences, University of Alicante, 03690 Alicante, Spain; rm.perez@ua.es

**Keywords:** nursing, working environment, personal protective equipment, personal well-being, SARS-CoV-2, COVID-19 pandemic

## Abstract

During the COVID-19 pandemic, nurses have had to face logistical problems related to critical changes in their work environment, the supply of personal protective equipment (PPE), and hard personal life decisions. This study aimed to investigate the changes in the working environment, PPE use, personal lives, and well-being as perceived by nurses in hospitals and primary care centers in Spain, during the COVID-19 pandemic. A descriptive cross-sectional survey study was conducted in April 2020, including 61 public and private hospitals, and 852 primary care centers. A total of 1296 nurses participated. The redeployment practice for organizational or vulnerability reasons was used by 26.4% of the participants. A total of 61.9% of the nurses doubled the time they used masks, and 8.3% of them could only replace the mask once a week. A total of 11.2% (*n* = 145) of the nurses left home to avoid infecting their family. Finally, 60.97% of the participants stated that their well-being was reduced compared to the state before the pandemic. Changes in the work environment, the use of PPE, and personal life may be related to the decrease in personal well-being perceived by the nurses.

## 1. Introduction

SARS-CoV-2 was first identified in Wuhan, China, in December 2019 [1,2], and it spread rapidly globally. The World Health Organization (WHO) declared on 11 March 2020, that the situation due to COVID-19 had changed from an epidemic to a pandemic [1]. On 31 January 2020, the first case of COVID-19 was diagnosed in Spain on La Gomera [3] and the first death was registered on 13 February in Valencia [4]. On 27 January 2022, the COVID-19 pandemic affected the world with 363,316,221 diagnosed cases and 5,628,898 deaths. Spain has become the country with the sixth highest number of affected populations (9,529,320) and 92,591 deaths [5]. In January 2022, the number of health professionals infected by COVID-19 totaled 116,207 in Spain. One of the regions with the most negative data has been the Valencian Community, with 18,278 health professionals infected (21.25% of the total in Spain) [6].

The affected countries had to reorganize hospitals and health centers to care for COVID-19 patients. In hospitals, recovery rooms, and operating rooms were converted into new Intensive Care Units, and these units were reinforced. It was necessary to hire health professionals who often lacked the required skills in these work environments. This critical situation led to the assumption of potential risks for patient care safety and quality [7].

Previous studies have shown that health professionals had to face logistical problems related to the supply of personal protective equipment (PPE) in affected countries [8,9]. Gloves, gowns, respiratory and eye protection, and mechanical ventilators were highly demanded [8]. The scarcity of materials and uncertainty in decision-making [9] diminished the attention to patients and their protection against possible contagions. Other studies have shown that compassion fatigue [10], post-traumatic stress [11], and moral damage in health professionals were linked to the intensity of the pandemic spread [12]. During the COVID-19 pandemic, health professionals suffered acute stress from caring for numerous critically ill patients [13]. As a result, health professionals’ physical and psychological safety had been at risk during this time [14,15,16,17].

In health systems’ organizational context, nurses are the backbone, being the most important number of health professionals [18] that work at the first line of patient care and, therefore, are exposed to higher viral loads [19,20]. The situation in the health system tested the security measures that nurses should carry out in their work.

In addition, nurses had an active role in protecting their families from the transmission of SARS-CoV-2, so they changed their behaviors and stayed in non-work or family environments [21,22].

The purpose of the study was to investigate the changes in the work environment, the use of PPE, and the changes in personal lives and well-being as perceived by nurses in hospitals and primary care centers during the COVID-19 pandemic, in the Valencian Community region.

## 2. Materials and Methods

### 2.1. Design, Setting and Population

**Figure 1:**
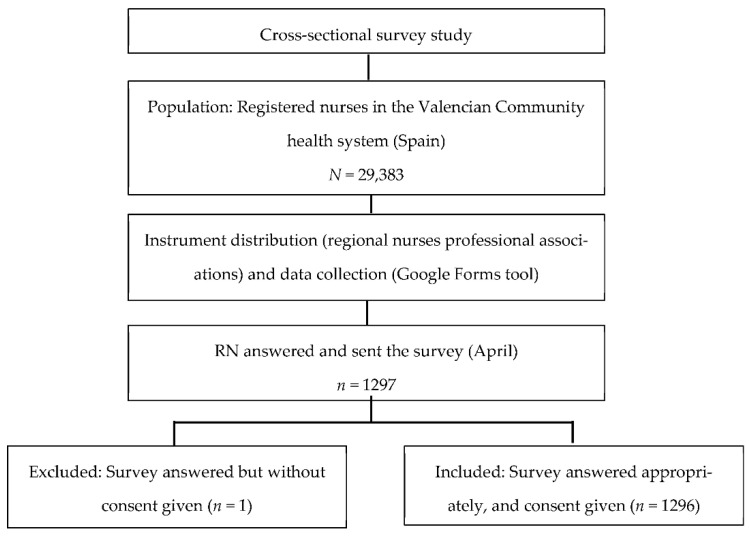
Flow diagram of the study.

The present descriptive cross-sectional survey study was conducted in a regional area of Spain, with a health system comprised of 61 public and private hospitals, and 852 primary care centers (Figure 1). The target population was the 29,383 registered nurses in the Valencian Community health system, who were invited to participate in the study through the nursing professional associations. A sample size of 1006 participants was estimated for a confidence level of 99% and an acceptable margin of error (4%). Inclusion criteria were nurses working in public or private health institutions in the Valencian Community at the time of the study. The exclusion criteria of the survey were to answer inappropriately or consent not given. Single-stage convenience sampling was planned for this exploratory study.

### 2.2. Instrument and Procedure

Based on scientific evidence and following the steps described by Boynton and Greenhalgh (study design, research questions, format, instructions, piloting, sampling, distribution) [23], a survey was designed to collect information on the work, social, and family factors affecting nurses. This instrument was entitled “Work and personal state of affairs of nurses in the COVID-19 pandemic”.

For this purpose, a questionnaire was designed with 22 items structured in four data blocks: (1) socio-demographic and work area (sex, age, academic qualification, category, and professional seniority); (2) work environment; (3) personal protective equipment (use and frequency of replacement); and (4) changes in the personal life of nurses. In addition, the questionnaire included two items to assess nurses’ perceptions about their well-being, perceived before and during the pandemic. A 10-point Likert scale was used to estimate well-being (from 0 = minimum expression of satisfaction to 10 = maximum expression of satisfaction).

An expert panel of three health professionals not linked to this research reviewed the survey. Their suggestions for improvement of the content and proposals for aspects to be included were collected. This review and evaluation contributed to the improvement and content validity of the designed instrument.

The nurses’ population of the Valencian Community were emailed a link to the survey developed using the Google Forms tool, with one reminder for completion one week apart. In addition, a WhatsApp broadcast list and social media networks (Twitter and Facebook) were used for the survey link distribution. The questionnaire was available online for ten days to reduce outcome bias at a specific point. Data were collected in April 2020.

### 2.3. Data Analysis

Statistical analyses of the variables were conducted using SPSS version 26 software (IBM Corp. Released 2019, Armonk, NY, USA). Continuous variables were abridged using descriptive statistics, including the number of observations used in the calculation (*n*), mean, and standard deviation (SD). In addition, categorical variables were summarized as counts and percentages of each category for the participants’ data. Well-being perception means (before and at the first wave of the COVID-19 pandemic) were compared with a *t*-test for one sample. *p*-values < 0.05 were statistically significant.

### 2.4. Ethical Considerations

The Ethics Committee of the University of Alicante approved this study (UA-2020-04-13). Informed consent was obtained from the participants, who were required to answer whether they were willing to participate in the questionnaire voluntarily and to subsequently confirm this before sending it.

### 2.5. Quality Appraisal

The authors followed the STROBE (STrengthening the Reporting of OBservational studies in Epidemiology) checklist for cross-sectional studies of EQUATOR (Enhancing the QUAlity and Transparency of Health Research) to provide quality to this study.

## 3. Results

### 3.1. Sample

A convenience sample of 1296 Spanish nurses joined the study. The response rate was 4.4% (the number of completed surveys divided by the number of nurses in the nursing professional associations of the region multiplied by 100). The participants’ description is shown in Table 1. The majority were female (87.3%, *n* = 1131), with an age range between 30–49 years old (64.1%, *n* = 831), and with nursing experience of more than 11 years (67.6%, *n* = 876). Most of them worked in public institutions (87%, *n* = 1128), and commonly their qualification was in general nursing (88.4%, *n* = 1144). The more frequent work areas of the participants were hospital wards (33%, *n* = 428), primary care centers (16.1%, *n* = 209), or intermediate/intensive care units (14%, *n* = 181).

### 3.2. Working Environment

The organizational changes in job positions or roles due to the COVID-19 pandemic were also studied. Formulated questions and their answers are shown in Table 2. A total of 16.7% (*n* = 217) of the nurses were relocated, and 9.7% (*n* = 126) were hired to reinforce. On one hand, 40.4% (*n* = 524) of the participants mentioned that the nurse–patient ratio was increased in their services, and 16.2% (*n* = 210) mentioned that it was decreased.

Nurses were questioned if they had needed to change their job position if they were vulnerable. The highest valid percentage of sick leave approved was due to pregnancy situations (40%, *n* = 6), followed by sick leave due to immunosuppression disorder (28.57%, *n* = 8), and chronic lung disease (23%, *n* = 9). The lowest valid percentage of sick leave approved was for age reasons (9.6%, *n* = 5). The nurses’ positions had already been changed by diabetes (55%, *n* = 11), immunosuppression disorder (42.86%, *n* = 12), cancer in active treatment (41.67%, *n* = 5), over 60 years (32.7%, *n* = 17), cardiovascular disease (31.15%, *n* = 19), and chronic lung disease (23%, *n* = 9).

### 3.3. Personal Protective Equipment

In this study, 56.7% (*n* = 735) of the nurses declared having received training in personal protective equipment, but 73.4% (*n* = 951) would like additional information and training. In addition, participants marked their satisfaction level with personal protection material from 0 to 10, and the mean was 4.85 (SD = 2.5).

Additionally, nurses were asked about the frequency of replacement and reuse of personal protective equipment (Table 3). The most common surgical mask replacement frequency was one per shift (61.9%, *n* = 802). A total of 8.3% of the participants stated that they replaced the surgical mask once a week, and 4.4% only did so when it deteriorated.

Moreover, 12.6 (*n* = 163) and 59.2% (*n* = 767) of nurses affirmed they have never used an FPP2 and FPP3 mask, respectively, as PPE in the workplace. A once-weekly replacement was reported by 14.7% for FPP2 and 4.9% for FPP3.

The participants stated having to reuse protective materials such as surgical masks (62.2%, *n* = 806) and FPP2 masks (70.1%, *n* = 908).

### 3.4. Changes in Nurses’ Personal Lives

Study participants were also asked about perceived changes in their personal life and well-being because of the COVID-19 pandemic (Table 4). Only 11.2% (*n* = 145) left home to avoid infecting their family. Most (87.6%, *n* = 127) moved to a second or empty family home. Most commonly, nurses’ children were overseen by their father/mother/partner (61.6%, *n* = 494) while they were working. In other cases, they stayed alone (19.1%, *n*= 153) or their grandparents (11.6%, *n* = 93) oversaw them.

Half of the participants that were the primary caregiver of a dependent person had to stop this role due to the contagion risk (50%, *n* = 114). It was also not easy to obtain shift changes for family conciliation. More than half of the nurses (64.6%, *n* = 445) were not given the opportunity or were not allowed to change shifts to reconcile with family life (Table 4).

### 3.5. Perceived Well-Being

**Figure 2:**
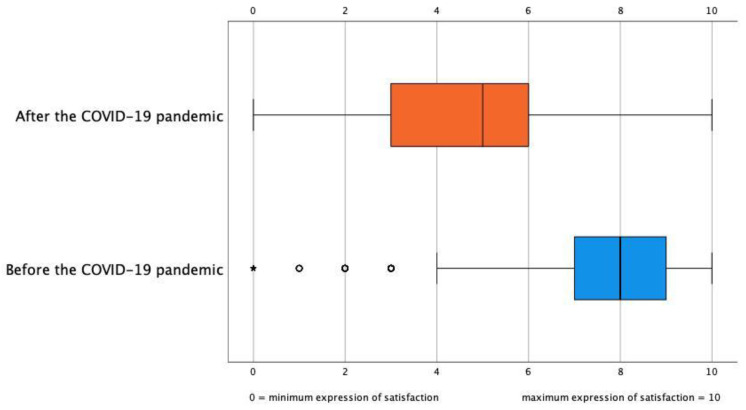
Well-being perception as a nurse before and at the COVID-19 pandemic first wave (*n* = 1296).

In the present study, participants were invited to grade their perceived well-being from 0 to 10 (Figure 2). They gave two marks: one for their well-being before the pandemic, and the second for their perceived well-being at that moment–the first wave of the COVID-19 pandemic. The participants’ mean well-being score as nurses was 7.61 (SD = 1.8) before the pandemic. However, the measurement’s mean score was 4.64 (SD = 2.2) coinciding with the pandemic’s first wave. The well-being perception means (before and at the first wave of the COVID-19 pandemic) were compared with a *t*-test for one sample, assuming a test value of 7.61 (well-being score before the pandemic). *T*-test was −48.61 (df = 1295), *p* < 0.001.

## 4. Discussion

This study describes the situation related to organizational changes in nurses’ job positions or roles during the COVID-19 pandemic in Spain, focusing on nurses’ perceptions of changes in the working environment, the use and replacement of PPE, and the changes in their personal lives and well-being.

### 4.1. Working Environment

The increase in patients requiring medical care with COVID-19-related symptoms has imposed unprecedented pressure in nurses’ work across the healthcare systems around the world. This situation implied organizational changes in the jobs or roles of the nurses. More than a quarter of nurses had changed their position or been hired to reinforce a new ward/unit in this study.

According to the Ministry of Health in Spain, the redeployment sought to increase the number of nurses in hospital units or health centers and relocate vulnerable professionals, according to the action procedure established [24]. Concerning this legislation, the study’s data show that a high percentage of vulnerable professionals had not yet been relocated when the survey was conducted. The highest percentage of vulnerable professionals without relocation were those over 60 years of age and nurses with cardiovascular diseases, even though the evidence shows that patients with COVID-19 and cardiovascular disease have a higher risk of dying [25], as also occurs with those over 60 years of age [26]. Likewise, a significant lack of relocation has also been observed in patients with chronic lung disease and cancer under active treatment, who also have a higher risk of death when sick with COVID-19 [27]. On the other hand, the most significant requests for change of ward or sick leave approval were in pregnant women and people with immunosuppression. Previous evidence was found assessing the vulnerability of employees of different occupations, including a sample of healthcare professionals during the COVID-19 pandemic in Iran [28]. However, the results did not focus on the pathologic status of these professionals.

In the authors’ opinion, the redeployment of professionals at risk effectively guaranteed the physical health of healthcare professionals; nevertheless, psychological vulnerability should also be considered. The increase in the nursing staff to the European ratio standards would have been a buffer factor in the saturation of care for patients with COVID-19 in hospitals and primary care centers. Nowadays, the European average is 8.8 nurses per 100,000 inhabitants; in Spain it is 5.3 and in the Valencian Community it is 4.79 [29]. In other words, the nurse–patient ratio in Spain and the Valencian Community is well below the European average.

### 4.2. Personal Protective Equipment

The lack of information and training for healthcare professionals is a substantial issue since the lack of training contributes to insecurity and more significant psychological distress in these professionals [30]. Most of the nurses (73.4%) in our study desired additional information and training on using PPE for COVID-19+ patient care. Our results are slightly higher than those reported in a study carried out in Brazil, Colombia, and Ecuador [31], which found that half of the healthcare professionals reported a lack of knowledge about PPE use. Moreover, our data are higher than those found by Tabah et al. [32], in which 49% of the participants desired additional training, despite 83% having stated full competence in using PPE. A possible explanation for the differences between studies’ results is the mixed composition of the sample. In our study, only nurses participated, while in the other studies [31,32] nurses, physicians, and other healthcare staff participated.

Previous studies have synthesized the recommendations for the use of PPE issued by institutions of recognized prestige, such as the World Health Organization, the European Center for Disease Prevention, the Center for Evidence-Based Medicine, or the Ministry of Health of the Government from Spain [33,34]. These indications are included in the UNE-EN 14683: 2019 + AC: 2019 regulation regarding surgical masks and the UNE-EN 149: 2001 + A1: 2010 regulation for Filtering Face Piece (FFP) masks. The regulations indicate that the application of the surgical mask limits the transmission of pathogens from the healthcare professional to patients during surgical interventions and decreases the spread of infectious agents through symptomatic/asymptomatic patients [34]. Furthermore, self-filtering masks (FFP1, FFP2, and FFP3) offer a superior level of protection for nurses’ airways, as they can filter smaller particles [35].

In this study, nurses used mainly surgical masks when dealing with COVID-19+ patients; despite the degree of protection provided they are not effective when performing aerosol-generating procedures on the patient, since the filter does not prevent the penetration of smaller particles. What is more, nurses negatively highlighted the reuse of both types of masks, arguing that they could not be replaced due to a lack of supplies at that time.

As the WHO states, the protection of first-line health workers is essential [36]. Surgical masks, FFP2, and FFP3 are recommended as single-use products [37], with a maximum wearing time for surgical masks set at 4 h, according to the French Society for Hospital Hygiene [38] or 6 h according to the WHO [36]. Nevertheless, the increased demand for PPE during the first wave of the pandemic led to an interruption in the supply of gloves, masks, and gowns [37] that was negative for health professionals worldwide. This study reflects the increase in the frequency of use of PPE, as well as its reuse, especially regarding masks. These results point to the difficulty in guaranteeing the safety of healthcare personnel during patient care. Previous studies assert that healthcare workers experienced high rates of infection and death, in part due to inadequate access to PPE [39]. It should be remembered that health professionals are at increased risk of contracting the disease due to their exposure to higher viral loads [40].

### 4.3. Changes in Nurses’ Personal Lives

Regarding family conciliation, the results show that 11.2% of the nurses left home for fear of infecting their family, and they had to delegate their care responsibilities to other members of their family structure. What is more, this fear was shared by the whole family. This emotional state is described by Stephanie Chandler-Jeanville [41] as a psychological vulnerability of families due to sharing the fear and anxiety of seeing how their healthcare relative could become infected by being more exposed to SARS-CoV-2 at work.

### 4.4. Perceived Well-Being

In our study, participants’ well-being before the pandemic was 7.61 (possible maximum = 10), and during the first wave, it was 4.64, which represented a reduction of 39.02%. In previous studies [32,42], the factors that influenced the well-being of nurses were analyzed in-depth, determining that the higher the number of patients, the more significant the workload, which increased the possibility of infection and physical exhaustion, which translated into a higher level of perceived stress and a poor quality of sleep.

Bellanti et al. [43] focused their research on nurses’ burnout and its associated factors in the hospital setting, obtaining very consistent results in burnout and exhaustion. Almost 90% of the participants met the medium/high burnout criteria. At the same time, 70% exhibited emotional exhaustion associated with emotional support, consideration of leaving work, workload, and stress. Significantly, burnout was not related to demographic characteristics or occupational factors, such as working in a COVID-19 patient unit or being directly exposed to infected patients in other units or services [43].

### 4.5. Limitations and Strengths

The study has some limitations that must be interpreted within its pandemic context and temporality. Due to its design’s nature, it has the drawback of providing data from a single moment in time and the weakness of a convenience sample. Furthermore, the data were only collected in one of the 17 regional areas of Spain, which makes it problematic to generalize the data to other regions or countries. However, it should be noted that the sample size adequately represented the population of nurses in the Valencian Community.

## 5. Conclusions

The current pandemic has presented unprecedented challenges, and difficulties have been encountered worldwide. This study sought to describe the situation and the main problems nurses faced during the COVID-19 pandemic. Over a quarter of the nurses had been redeployed to reinforce services such as ICU, emergency, and hospitalization. Nurses over the age of 60 years and those with cardiovascular disease, chronic lung disease, or cancer under active treatment had not been relocated for the most part at that time. Pregnant nurses and those with immune disorders were already relocated or had sick leave approval. Three out of four nurses stated that they were not sufficiently trained in PPE use. Participants reported the need to reuse PPE material due to limited supplies. The shortage of material caused the extension in using the surgical mask and the FFP2/FPP3 mask. Fortunately, the percentage of nurses who left their homes for fear of infecting their family was not very high, but the situation meant that the family care of minor children was delegated mainly to their partners or to grandparents. For the participants, their well-being had decreased considerably in a short time.

It would be recommendable to understand and learn from what happened to reverse the general situation of exhaustion associated with workload, lack of training and materials, alteration in family reconciliation, and changes in personal well-being. Health managers and governments should implement in-depth changes and attend to nurses’ physical and psychological health.

## Figures and Tables

**Table 1:** Participants’ description (*n* = 1296).

	*n*	%
**Gender**		
Female	1131	87.3
Male	165	12.7
**Age**		
22–29	205	15.8
30–39	423	32.6
40–49	408	31.5
50–59	207	16
≤60	53	4.1
**Years as nurses**		
0–5	252	19.4
6–10	168	13.0
11–15	378	29.2
16–20	240	18.5
More than 20	258	19.9
**Type of institution**		
Public institutions	1128	87
Private institutions	168	13
**Nursing professional group**		
General nursing (without specialty)	1144	88.4
Pediatric Nurse Specialist	65	5.1
Midwifery	42	3.2
Family and Community Nurse Specialist	16	1.2
Mental Health Nurse Specialist	11	0.8
Occupational Health Specialist Nurse	11	0.8
Geriatrics Nurse Specialist	7	0.5
**Working area**		
Hospital ward	428	33
Primary care	209	16.1
Intermediate or Intensive Care Unit	181	14
Other hospital units	125	9.6
Emergencies	123	9.5
Operating rooms	80	6.2
Outpatients’ departments	53	4.1
Other workplaces	31	2.4
Delivery rooms	22	1.7
Socio-health community centers	28	2.2
Home hospitalization unit	16	1.2

**Table 2:** Organizational changes in job position or role as a nurse: redeployment (*n* = 1296).

What Has Happened to Your Job?		*n*	%	
I am still in the same job		953	73.5	
I have been relocated		217	16.7	
I have been hired to reinforce		126	9.7	
**What has happened to the nurse–patient ratio in your service**?				
The nursing staff has been increased		524	40.4	
It keeps the same nursing staff		524	40.4	
The nursing staff has been decreased		210	16.2	
**If you are vulnerable personnel, have you changed your position or role? Have you been redeployed?**		***n***	**%**	**Valid percentage ^1^**
Pregnancy	Yes ^2^	5	0.4	33.33
	Not yet	4	0.3	26.66
	Sick leave approved	6	0.5	40
	I do not meet the criteria	1281	98.8	-
Immunosuppression disorder	Yes	12	0.9	42.86
	Not yet	8	0.6	28.57
	Sick leave approved	8	0.6	28.57
	I do not meet the criteria	1268	97.8	-
Cardiovascular disease	Yes	19	1.4	31.15
	Not yet	35	2.7	57.38
	Sick leave approved	7	0.5	11.47
	I do not meet the criteria	1235	95.3	-
Diabetes	Yes	11	0.8	55
	Not yet	7	0.5	35
	Sick leave approved	2	0.2	10
	I do not meet the criteria	1276	98.5	-
Chronic lung disease	Yes	9	0.7	23
	Not yet	21	1.6	54
	Sick leave approved	9	0.7	23
	I do not meet the criteria	1257	97	-
Cancer in active treatment	Yes	5	0.4	41.67
	Not yet	5	0.4	41.67
	Sick leave approved	2	0.2	16.66
	I do not meet the criteria	1284	99.1	-
Over 60 years	Yes	17	1.3	32.7
	Not yet	30	2.3	57.7
	Sick leave approved	5	0.4	9.6
	I do not meet the criteria	1244	96	-

^1^ For the sample that meets the criteria. ^2^ Yes, I have been redeployed.

**Table 3:** Frequency of replacement and reuse of personal protective equipment (*n* = 1296).

Frequency of Replacement								Reuse	
	Once Per Shift *n* (%)	Once per Week *n* (%)	After Contact with COVID-19 +/Possible COVID-19 Patient *n* (%)	Only when It Deteriorates *n* (%)	Never *n* (%)	Other *n* (%)	Yes *n* (%)	No *n* (%)	I Have Never Had *n* (%)
Surgical mask	802	108	274	57	3	52	806	466	24
	(61.9)	(8.3)	(21.1)	(4.4)	(0.2)	(4)	(62.2)	(36)	(1.8)
FPP2 mask	303	191	366	178	163	95	908	208	180
	(23.4)	(14.7)	(28.2)	(13.7)	(12.6)	(7.3)	(70.1)	(16)	(13.9)
FPP3 mask	65	63	214	60	767	127	287	173	836
	(5)	(4.9)	(16.5)	(4.6)	(59.2)	(9.8)	(22.1)	(13.3)	(64.6)
Safety glasses	185	35	663	91	200	122	873	177	246
	(14.3)	(2.7)	(51.2)	(7)	(15.4)	(9.4)	(67.4)	(13.6)	(19)
Face shield	242	42	580	139	150	143	938	206	152
	(18.7)	(3.2)	(44.8)	(10.7)	(11.6)	(11)	(72.4)	(15.9)	(11.7)
Waterproof gown	232	36	607	66	216	139	566	468	262
	(17.9)	(2.8)	(46.8)	(5.1)	(16.7)	(10.7)	(43.7)	(36.1)	(20.2)
Surgical gown	375	54	479	58	134	196	490	643	163
	(28.9)	(4.2)	(37)	(4.5)	(10.3)	(15.1)	(37.8)	(49.6)	(12.6)
Full-body isolation suit	113	38	398	38	549	160	233	379	684
	(8.7)	(2.9)	(30.7)	(2.9)	(42.4)	(12.3)	(18)	(29.2)	(52.8)

**Table 4:** Changes in nurses’ personal lives due to the COVID-19 pandemic.

Have You Left Your Home to Avoid Infecting Your Family? (*n* = 1296)	*n*	%
Yes	145	11.2
No	1151	88.8
**After leaving your usual home, where have you stayed? (*n* = 145)**		
Second residence or empty family home	127	87.6
I have paid for a hotel/flat/apartment	14	9.6
In hotel/flat/apartment made available altruistically by a company	4	2.8
**If you have children, who has overseen them while you were working? (*n* = 802)**		
Father/mother/my partner	494	61.6
They stay alone (by age)	153	19.1
Grandparents	93	11.6
Other family and friends	31	3.9
Paid staff	21	2.6
In turn, both parents are healthcare professionals	10	1.2
**If you are the primary caregiver of a dependent person, have you stopped doing this to avoid contagion? (*n* = 228)**		
Yes	114	50
No	114	50
**Have they facilitated shift changes for family conciliation in your job? (*n* = 689)**		
Yes	219	31.8
No	445	64.6
I had to request an extension of reduced working hours	25	3.6

## Data Availability

The data presented in this study are available on request from the last author.
